# A randomized controlled trial evaluating the effectiveness of an acceptance and commitment therapy–based bibliotherapy intervention among adults living with chronic pain

**DOI:** 10.1080/24740527.2019.1678113

**Published:** 2019-11-26

**Authors:** Josée Veillette, Marie-Eve Martel, Frédérick Dionne

**Affiliations:** a Department of Psychology, Université du Québec à Trois-Rivières, Trois-Rivières, Quebec, Canada; b Quebec and Pain Research Network (QPRN), Montreal, Quebec, Canada

**Keywords:** chronic pain, self-help, acceptance and commitment therapy (ACT), pain-related disability, psychological inflexibility

## Abstract

**Background**: Chronic pain has a significant impact on the physical and psychological functioning of those living with this condition. It is now recognized that acceptance and commitment therapy (ACT) is an effective intervention in managing chronic pain; however, several barriers limit its accessibility.

**Aims**: The current study aimed to evaluate the effectiveness of an 8-week bibliotherapy-type self-administered psychological intervention with minimal therapeutic contact, based on ACT, in the management of chronic pain.

**Methods**: This was a randomized controlled trial with three measurement periods (pretest, posttest, and 3 months after the intervention; ClinicalTrials.gov Identifier: NCT03924687). A total of 140 adults with chronic pain were randomly assigned to an ACT self-help condition or a wait-list control condition.

**Results**: Two-way repeated measures analysis of variance (ANOVA) models showed statistically significant differences between pretest and posttest in terms of pain-related disability (main variable), depression (secondary variable), pain-related acceptance, and psychological inflexibility (*d* = 0.46–0.88) in favor of the ACT self-help condition. At the 3-month follow-up, these differences were maintained and nearly 54% of participants reported an overall improvement of their physical and mental health.

**Conclusion**: These results suggest that a psychological intervention self-administered through ACT bibliotherapy with minimal therapeutic support can improve the physical and emotional functioning of adults from the community who live with chronic pain.

## RÉSUMÉ

**Contexte:** La douleur chronique a des répercussions importantes sur le fonctionnement physique et psychologique des personnes atteintes. Il est maintenant reconnu que la thérapie d'acceptation et d'engagement (ACT) est une intervention efficace pour la prise en charge de la douleur chronique; cependant, plusieurs barrières limitent son accessibilité.

**Objectifs:** La présente étude avait pour but d’évaluer l'efficacité d'une intervention psychologique autoadministrée de huit semaines sous forme de bibliothérapie avec contact thérapeutique minimal, basée sur l'ACT dans la gestion de la douleur chronique.

**Méthodes:** Cet essai contrôlé et randomisé comprenait trois temps de mesure (prétest, posttest et trois mois suivant l’intervention) (Code d’identification ClinicalTrials.gov :NCT03924687). Un total de 140 adultes souffrant de douleur chronique a été assigné aléatoirement dans une condition ACT de type self-help (bibliothérapie) et une condition liste d'attente.

**Résultats:** Un modèle d'analyse de variance (ANOVA) à deux facteurs à mesures répétées a démontré des différences statistiquement significatives entre le prétest et le posttest sur l'incapacité liée à la douleur (variable principale), la dépression (variable secondaire), l'acceptation et l'inflexibilité psychologique associées à la douleur (d = 0,46 à 0,88) en faveur de la condition ACT de type self-help. Au suivi de trois mois, ces différences se sont maintenues et près de 54 % des participants ont rapporté une amélioration globale de leur santé physique et mentale.

**Conclusion:** Ces résultats suggèrent qu'une intervention psychologique autoadministrée par le biais d'une bibliothérapie de type ACT, avec soutien thérapeutique minimal, peut améliorer le fonctionnement physique et émotionnel des adultes souffrant de douleur chronique en provenance de la communauté.

## Introduction

Over the last 30 years, psychological approaches based on acceptance and mindfulness have emerged in the field of chronic pain (CP).^1,^^2^ Acceptance and commitment therapy (ACT)^3,4^ is an approach that stemmed from this flow of contemporary cognitive and behavioral therapies. The main purpose of ACT is to develop greater psychological flexibility. Six therapeutic processes underlie its theoretical model (called the psychological in/flexibility model): acceptance, cognitive defusion, mindfulness, self-as-context, values, and committed action. In the context of pain, psychological flexibility refers to openness toward unpleasant painful experiences of physical and psychological symptoms linked to CP, the reduction of unsuccessful attempts to control or avoid the pain, and commitment to valued activities likely to enrich one’s quality of life.^2,5^ Empirical support for ACT in the management of CP has increased considerably over the last few years. The results of a meta-analysis^6^ suggest that ACT can significantly improve the physical (e.g., pain-related disability) and psychological (e.g., depression) functioning of people with CP, with varying effect sizes, ranging from weak to moderate.

Nevertheless, several barriers limit accessibility to empirically validated interventions in the management of CP: an insufficient number of specialists qualified and trained in these approaches, long wait lists, costs linked to the intervention, the remoteness of participants from large urban centers, difficulties linked to their mobility or transportation, and their reluctance to accept face-to-face treatment because of the prejudice or stigma that may be associated with the use of such services.^7–9^ Given these numerous obstacles, self-help psychological interventions have received growing attention from researchers over the last few decades. These standardized psychosocial interventions can be administered from a book (bibliotherapy), Web platforms, or mobile applications and are offered according to various levels of therapeutic support.^10–12^ A growing number of studies support the use of ACT-based, self-help approaches for various behavioral, psychological, and physical issues, including CP. A meta-analysis and systematic review by French et al.,^13^ including 13 randomized controlled trials (RCTs) and totaling 2580 participants, showed small but significant effect sizes on anxiety, depression, and psychological flexibility; however, studies related to self-help interventions based on ACT in the management of CP among adults remain limited.

A few RCTs evaluated interventions for chronic pain in the form of bibliotherapy,^14,15^ a Web platform,^16–18^ or a smartphone via a mobile application^19^ compared to expressive writing,^18^ traditional cognitive and behavioral therapy,^15^ a discussion forum,^16^ a noninteractive website,^19^ and a wait list.^14,17^ Overall, the results of these interventions show significant improvements in favor of ACT for physical functioning (pain-related interference/disability),^15,17,18^ pain intensity,^15,16,18^ mood,^14,16,17^ life satisfaction,^15^ quality of life,^14^ and pain catastrophizing^19^ and for the processes linked to the therapeutic model of ACT.^15–19^ In general, these results are maintained during longer term follow-ups.^15–19^

Although these results seem promising, psychological self-help pain management interventions, including ACT, have certain limitations. Research indicates that the results and effect sizes for pain-related disability and mood vary considerably.^20^ Furthermore, literature generally considers that pain relief is accompanied by functional improvement. That said, studies have demonstrated that pain intensity and physical functioning are only modestly correlated,^21^ which is why it is relevant to consider pain-related disability as the main outcome variable, rather than pain intensity.^22,23^ Several authors also mention the importance of evaluating the impact on the life of the patient over the longer term (improvement or worsening of their condition). The IMMPACT (Initiative on Methods, Measurement, and Pain Assessment in Clinical Trials) expert consensus^24^ in the treatment of CP suggests using the Patient Global Impression of Change (PGIC)^25^ scale to measure treatment effect.^26,27^

In studies evaluating self-administered ACT to manage CP, acceptance is the process most studied. Past research has recommended the inclusion of more components linked to its therapeutic model.^28^ Systematic evaluation of the ACT psychological flexibility model variables would help to adapt the interventions to the specific needs of those suffering from CP, improve clinical results, and further support the psychological flexibility model in the field of CP.^29^ At present, only one study^18^ used a pain-related psychological inflexibility measure, characterized by the fusion of thoughts and behavioral avoidance.^30^ Furthermore, to our knowledge, only two RCTs have evaluated the effectiveness of an ACT intervention program that is self-administered as bibliotherapy, and these trials have shortcomings. For example, the study conducted by Thorsell et al.^15^ included many telephone contacts with a clinician, making it harder to evaluate effects specifically related to reading the book. The study by Johnston et al.^14^ recruited a very small sample (*N* = 14), and the treatment effects over the long term were not examined. This limited number of RCTs does not support the conclusion that bibliotherapy based on ACT is effective in the management of CP specifically. Furthermore, the effectiveness of self-help books based on ACT available to the general public have been subject to various criticisms regarding the overestimation of the effectiveness of the types of intervention.^31–33^

Given the advantages of self-help programs (e.g., low cost, high accessibly) and the gaps in the current literature, it is crucial to conduct further studies, using larger samples, to better examine the effect of this type of intervention for people who live with chronic pain. The purpose of this randomized controlled trial was to assess the effectiveness of an 8-week, ACT-based, self-administered bibliotherapy intervention program with minimal therapeutic support in the management of CP.

This study was based on the following hypotheses. In comparison to the wait-list control group, from pre to post, the ACT self-help program will show significantly

- lower pain-related disability (primary variable);
- improved depressive symptoms related to CP (secondary variable);
- increased pain acceptance scores (process variable);
- less psychological inflexibility linked to painful symptoms (process variable).

It was also expected that

- the improvements would be maintained at a 3-month follow-up; and
- self-help participants would have an overall impression of a positive change following the intervention.

## Methods

The present study was approved by the Ethics Committee for Research on Human Subjects of the Université du Québec à Trois-Rivières (certificate number: CER-15-215-07.23), and ethical standards of the Canadian Psychological Association were followed. The study’s protocol, *Evaluating the Effectiveness of an ACT-Based Bibliotherapy Intervention Among Adults Living with Chronic Pain*, has been registered at ClinicalTrials.gov (Identifier: NCT03924687). CONSORT 2010 guidelines for randomized trials were followed (see Table 1).^34^

**Table 1.** CONSORT 2010 guidelines for randomized trials.

Section/topic	Item no.	Checklist item	Reported on page:
Title and abstract			
	1a	Identification as a randomized trial in the title	1
	1b	Structured summary of trial design, methods, results, and conclusions (for specific guidance see CONSORT for abstracts)	1
Introduction			
Background and objectives	2a	Scientific background and explanation of rationale	1–4
	2b	Specific objectives or hypotheses	4–5
Methods			
Trial design	3a	Description of trial design (such as parallel, factorial) including allocation ratio	5
	3b	Important changes to methods after trial commencement (such as eligibility criteria), with reasons	n/a
Participants	4a	Eligibility criteria for participants	6
	4b	Settings and locations where the data were collected	6
Interventions	5	The interventions for each group with sufficient details to allow replication, including how and when they were actually administered	7–8, Table 2
Outcomes	6a	Completely defined prespecified primary and secondary outcome measures, including how and when they were assessed	7–11
	6b	Any changes to trial outcomes after the trial commenced, with reasons	n/a
Sample size	7a	How sample size was determined	5–6
	7b	When applicable, explanation of any interim analyses and stopping guidelines	n/a
Randomization:			
Sequence generation	8a	Method used to generate the random allocation sequence	6
	8b	Type of randomization; details of any restriction (such as blocking and block size)	6
Allocation concealment mechanism	9	Mechanism used to implement the random allocation sequence (such as sequentially numbered containers), describing any steps taken to conceal the sequence until interventions were assigned	n/a
Implementation	10	Who generated the random allocation sequence, who enrolled participants, and who assigned participants to interventions	6
Blinding	11a	If done, who was blinded after assignment to interventions (for example, participants, care providers, those assessing outcomes) and how	n/a
	11b	If relevant, description of the similarity of interventions	n/a
Statistical methods	12a	Statistical methods used to compare groups for primary and secondary outcomes	11–13
	12b	Methods for additional analyses, such as subgroup analyses and adjusted analyses	n/a
Results			
Participant flow (a diagram is strongly recommended)	13a	For each group, the numbers of participants who were randomly assigned, received intended treatment, and analyzed for the primary outcome	13–14, Figure 1
	13b	For each group, losses and exclusions after randomization, together with reasons	Figure 1
Recruitment	14a	Dates defining the periods of recruitment and follow-up	6–7
	14b	Why the trial ended or was stopped	n/a
Baseline data	15	A table showing baseline demographic and clinical characteristics for each group	Table 3
Numbers analyzed	16	For each group, number of participants (denominator) included in each analysis and whether the analysis was by original assigned groups	Tables 4 and 6
Outcomes and estimation	17a	For each primary and secondary outcome, results for each group and the estimated effect size and its precision (such as 95% confidence interval)	15–17 Tables 4–6
	17b	For binary outcomes, presentation of both absolute and relative effect sizes is recommended	n/a
Ancillary analyses	18	Results of any other analyses performed, including subgroup analyses and adjusted analyses, distinguishing prespecified from exploratory	n/a
Harms	19	All important harms or unintended effects in each group (for specific guidance see CONSORT for harms)	11, Table 7
Discussion			
Limitations	20	Trial limitations, addressing sources of potential bias, imprecision, and, if relevant, multiplicity of analyses	20–22
Generalizability	21	Generalizability (external validity, applicability) of the trial findings	22
Interpretation	22	Interpretation consistent with results, balancing benefits and harms and considering other relevant evidence	18–20
Other information			
Registration	23	Registration number and name of trial registry	5
Protocol	24	Where the full trial protocol can be accessed, if available	On clinical trials.gov
Funding	25	Sources of funding and other support (such as supply of drugs), role of funders	23

### Research design

This study was a randomized controlled trial with three measurement periods (pretest, posttest, and 3 months postintervention) thereafter to compare the effects of the ACT self-help program with a wait-list control group.

### Sample size estimation

The randomized trials evaluating ACT-based self-help interventions for CP report effect sizes of *d* = 0.33,^18^ *d* = 0.56,^16^ and *d* = 0.58^17^ for pain-related disability immediately following the intervention compared to a wait-list condition. Consequently, this study considered a minimal effect size of *d* = 0.4 as the clinical significance threshold for pain-related disability (main variable). Assuming a statistical power of 80% with a significance level of *P* = 0.05,^17,18^ the total sample had to include at least 125 people, given a possible attrition rate of 30%^35^ between the pretest (T1) and posttest (T2).

### Randomization

Participants were randomly selected and assigned by one of the researchers to the ACT self-help or wait-list control conditions using a simple randomization method (a random number list generated by Excel).

### Participants

Study participants were recruited between March 8 and March 13, 2016 (inclusive), with the help of the Association québécoise de la douleur chronique, an organization composed of people who suffer from CP in Quebec (Canada). Selection criteria were the following: (1) being 18 years of age or older; (2) having suffered from daily pain for more than 3 months; (3) having reading and writing abilities in French equivalent or superior to grade 8; (4) having access to Internet at home and having a valid e-mail address; (5) not having previously completed an ACT-type psychotherapy, not having practiced mindfulness meditation regularly, and not having read a bibliotherapy on ACT for pain; and (6) having stable medication for at least 1 month, if applicable.

### Recruitment procedure

Members of Association québécoise de la douleur chronique received an e-mail informing them that our research team was looking for adults suffering from CP willing to participate in an 8-week bibliotherapy-type psychological intervention program. Following this e-mail, interested individuals were automatically directed to a secure website to fill out a screening questionnaire to determine their eligibility. If eligible, they were redirected to a website containing an information and consent form that candidates were asked to read and sign electronically.

### Intervention

#### ACT self-help condition

The week of March 14, 2016, a first phone call was made to inform participants of the ACT self-help group about the experimental procedures and program terms and conditions. Though they had previously obtained study information through the online information and consent form, this was an opportunity for them to establish contact with the research assistant and ask questions about the study. They were then invited to complete a first series of baseline questionnaires (T1) via a secure website. The intervention itself started the week of March 28, 2016, following the completion of T1 measures, and ended the week of May 23, 2016. Each week of the intervention, participants received an e-mail presenting the week’s assigned activities; that is, readings, written exercises, meditation exercises to complete, etc. Midway through the intervention, a second phone call was made to answer questions regarding the various intervention tools, to support participants, and to foster adherence to the treatment.^36^ The study protocol anticipated a maximum duration of 15 min for each phone call (for both first and second phone calls). Participants could reach the intervention team by e-mail at all times. Doctoral students in psychology were available to answer questions and comments and offer psychological support if needed. These students also made the phone calls. Interventions (phone calls, responses to e-mails from participants, etc.) were supervised by a certified ACT psychologist. The team also provided technical support if necessary. The intervention protocol is presented in Table 2.

**Table 2.** Intervention protocol.

Week	Modules	Themes
0	—	Phone call
1	Module 1	Psychoeducation on chronic pain, introduction to ACT Introduction chapters, 1, 2 and 3
2	Module 2	Pain control (struggle), mindfulness Chapters 4 and 5
3	Module 3	Acceptance, committed action Chapters 6 and 7
4	Module 4	Acceptance (open arms) Chapter 8
5	Break	Phone call
6	Module 5	Cognitive defusion, self-as-context Chapters 9 and 10
7	Module 6	Values Chapter 11
8	Module 7	Pacing, mindfulness, and willingness to feel emotions Chapters 12 and 13
9	Module 8	Medication and insomnia through the ACT lens and concluding remarks Chapters 14, 15, 16

ACT = acceptance and commitment therapy.

#### Wait-list control condition

Like the ACT self-help group, participants of the wait-list control group received a phone call the week of March 14, 2016, to inform them of the experimental procedures and program terms and conditions and to invite them to complete the first series of questionnaires (T1). Participants in this condition were also invited to complete questionnaires after the 8-week period (T2). For ethical reasons, the wait-list control group received the intervention following completion of questionnaires at T2 but without completing any further follow-up measures.

### Intervention material

This 8-week program was based on the book titled *Libérez-vous de la douleur par la méditation et l’ACT*.^37^ It included the following components: (1) a participant’s workbook including written exercises to complete during the intervention (e.g., chart on which to log thoughts, meditation journals); (2) a website from which meditation exercises could be downloaded; (3) automated weekly e-mails; and (4) two phone calls of approximately 15 min each. Depending on the participant’s personal involvement, 1 to 4 h per week was required to complete the program activities, including T1 and T2 questionnaires.

### Measurement tools

To evaluate the impact of CP and the active processes of ACT, self-report scales measuring pain-related disability (main variable), depressive symptoms (secondary variable), pain acceptance, pain-related psychological inflexibility (variables linked to the ACT processes), and the participant’s general perception of treatment were administered. The measurement tools were selected based on the recommendations of the IMMPACT expert group.^24^

#### Sociodemographic and clinical information

This questionnaire included 12 items and helped obtain information related to participants’ sociodemographic characteristics (age, sex, ethnicity, education level, occupational status, living situation, family income), experience of pain (e.g., duration, pain diagnosis, use of pain relief medication), and other clinically relevant information (presence of a psychological disorder, type, and engagement in psychotherapy for pain). An additional item was added to evaluate pain intensity on average in the past 7 days on a scale from 0 (*no pain*) to 10 (*unbearable pain*). This questionnaire was administered at T1.

#### Primary outcome variable

##### Pain-related disability

Pain-related disability was evaluated using the modified 10-item version of the Interference subscale of the Brief Pain Inventory (BPI).^38–40^ This subscale evaluates to what extent pain disrupted various aspects of the person’s life over the last 7 days (general activity, mood, ability to walk, work, relationships with others, sleep, enjoyment of life, personal care, recreational activities, social activities). Items are evaluated on an 11-point Likert-type scale ranging from 0 (*does not interfere*) to 10 (*completely interferes*), with a maximum score of 100. A high score reveals increased functional disruption. The internal consistency of this subscale is estimated at α = 0.89.^40^ In this study, the alpha coefficient obtained was 0.90.

#### Secondary outcome variable

##### The Beck Depression Inventory

The short form of the Beck Depression Inventory (BDI-SF)^41^ was used to measure the depressive symptomatology according to 13 items. For each item, the respondents had to choose between four statements describing different levels of depressive symptoms over the last 7 days. Ratings varied from 0 to 3 for each item. Scores ranged from 0 to 39, with higher scores reflecting higher levels of depressive symptoms. For the short form, scores between 0 to 4 reflect the absence of depression, scores between 4 to 7 suggest light symptoms of depression, scores between 8 to 15 suggest the presence of moderate depression, and scores of 16 and above indicate severe depression. The BDI-SF has an internal consistency coefficient of 0.90. In this study, the alpha coefficient was estimated at 0.83. The test–retest procedure, with a 4-month interval, indicates good temporal stability (*r* = −0.62, *P* < 0.001). Just like the original version, the factorial analysis reveals three distinct components of depression: behavioral, cognitive, and somatic. Although this scale was not specifically designed to measure the intensity of depression among people with CP, the IMMPACT group recommends using it to measure their psychological and emotional functioning in clinical trials.^24^

#### Variables linked to ACT therapeutic processes

##### Pain acceptance

Pain acceptance was measured with the shorter form of the Chronic Pain Acceptance Questionnaire (CPAQ-8).^42^ The CPAQ-8 is an eight-item measure that evaluates acceptance of pain according to two subscales: Activity Engagement and Pain Willingness.^5,43^ The items are rated on a Likert-type scale from 0 (*never true*) to 6 (*always true*). Total scores range from 0 to 48 and a higher score reflects greater acceptance of pain. This short version of the CPAQ was validated with an online sample. It has an internal consistency of 0.85 to 0.89. In the current study, the alpha coefficient was equivalent to 0.76, which is deemed acceptable.

##### Psychological inflexibility in pain

Psychological inflexibility in pain was measured with the Psychological Inflexibility in Pain Scale (PIPS).^30^ This measure is composed of 12 items that evaluate two dimensions: avoidance and cognitive fusion. A Likert scale of 1 (*never true*) to 7 (*always true*) is used to estimate the level of inflexibility associated to pain. The scores range from 12 to 84, with higher scores revealing greater psychological inflexibility. This measure is consistent internally both in its original form (Cronbach alpha = 0.87) and the French version (α = 0.89).^44^ The alpha coefficient for this study is 0.84.

#### Supplementary variable linked to the follow-up

##### Patient Global Impression of Change

This five-item tool evaluates the level of change perceived by the participant regarding his or her pain, physical functioning, quality of life, and psychological well-being over the last 3 months.^25^ The statements are evaluated according to a 7-point Likert scale (e.g., “Over the last 3 months, your functioning 0) has deteriorated considerably; 3) has remain unchanged or 6) has improved considerably”). The scores were then recoded into three categories: (1) deteriorated, (2) unchanged, and (3) improved for each subscale. A fifth item evaluates pain relief over the last 3 months and is measured according to an ordinal scale ranging from 0 (*no relief*) to 100 (*total relief*). Although it is widely used in clinical trials on CP, the validity of this scale has not been officially evaluated.^45^ Its use is indicated by the IMMPACT expert group. This measure was only administered at follow-up (T3).

##### Negative effects and adherence to treatment

In order to account for potential negative effects associated with the intervention, three items were included for the experimental condition at T2 (e.g., “Certain exercises brought me negative side effects”). Items were evaluated on a 5-point Likert scale ranging from 0 (*strongly disagree*) to 4 (*strongly agree*). In order to measure adherence to treatment, five questions were also included at this time point (e.g., “In which proportion have you read the book?”) and participants were asked to rate their participation from 0% to 100%.

### Statistical analyses

To reduce the potential bias of the effects of the treatment stemming from missing data, all participants who completed measures at T1 were considered in the data analysis following the intention-to-treat principle. This analysis strategy helps to maintain the integrity of the randomization process and provides a more realistic estimate of the effects of the intervention,^46^ given that dropout risk and lack of adherence to treatment protocol are relatively common as part of self-help psychological interventions. Furthermore, the intention-to-treat analysis helps to maintain the size of the sample in order to prevent a reduction in statistical power.^47^ To do this, the missing data were processed using the last observation carried forward method. This method assumes that the score of participants who only completed the pretest evaluations (T1) would remain at least equivalent during the posttest (T2). As such, for the statistical analyses, the last measure available for each individual was transposed to the second measurement time to avoid overestimating the results.

The statistical analyses were conducted using the Statistical Package for the Social Sciences (SPSS, version 24). The normality postulate was verified by visual inspection of Q–Q plots and they were all normal. The Levene and Box *M* tests were used to assess the homogeneity of the variances and covariances of the model (*P >* 0.05). Overall, these requirements have been met, except for the BDI-SF, for which proper considerations were undertaken (see Results section). To evaluate the effects of the intervention, a two-way analysis of variance (ANOVA) model with a repeated measure on one factor (time) was used to compare the evolution of both groups between the first and second measurement times for each dependent variable studied. Preliminary analyses (single-factor ANOVA) confirmed the equality of both the ACT self-help program and wait-list control groups at baseline (T1), with no significant differences expected between means for each studied variable, except for the CPAQ-8 scale (see Results section). The level of statistical significance of ANOVAs was fixed at *P* ≤ 0.017 (.05/3) given the completion of multiple statistical tests, allowing for the analysis of the interaction effects and simple effects linked to time and condition (*P* ≤ 0.05/3 comparisons) to reduce the risk of a type 1 error (α).^48^ To estimate the magnitude of the effects obtained, the η^2^_partial_ was reported. According to Cohen,^49^ an effect size equal to ±0.01 is considered small, an index of ±0.06 is deemed moderate, and a η^2^_partial_ of more than 0.14 is considered large. To facilitate comparison with other studies, equivalence in terms of Cohen’s *d*s were also reported. A *d* with a value of 0.2 is considered a small clinical effect, an index of 0.5 is a medium effect size, and a value of more than 0.8 is a large effect size.^49^

Given the absence of a control group at 3 months postintervention (T3), Student’s *t* tests for paired samples were conducted to estimate the mean differences between T2 and T3 for the ACT self-help group. Normality of the distributions evaluated by the Shapiro-Wilk test was reached. The significance threshold was fixed at *P* ≤ 0.05. The Cohen’s *d* calculation was used to measure effects size. To avoid biases and better estimate the evolution of the participants and the actual effect at 3 months and given that nearly 50% of participants from the ACT self-help group did not complete measures at the 3-month follow-up, the analyses between T2 and T3 were conducted on the nonimputed observed data of the sample (*n* = 34).

## Results

### Participant flow throughout the study

**Figure 1.**
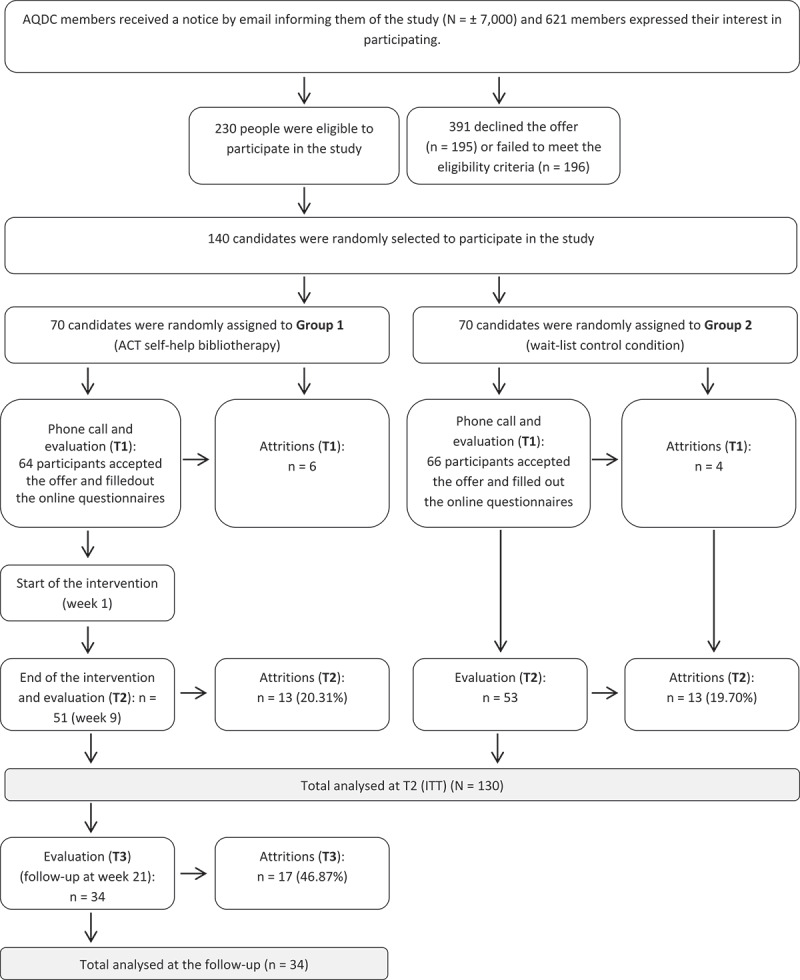
Participant flow throughout the study.

Among the 230 individuals who were interested in participating in the study and met the inclusion criteria, 140 candidates were randomly selected to ensure the minimum number of participants for the pretest (T1) and assigned to the ACT self-help (*N* = 70) or wait-list conditions (*N* = 70). By completing this first series of self-report questionnaires at T1, they were formally registered in the study. In total, 130 individuals (ACT self-help group: *n* = 64; wait-list control group: *n* = 66) completed these questionnaires. At the end of the program and wait-list period (week of May 23), participants received an e-mail asking them to complete a second series of online questionnaires (posttest; T2). To estimate the effects of the program in the longer term, participants in the ACT self-help group were asked to complete a third series of questionnaires 3 months postintervention (T3), the week of August 15, 2016. Eighty percent of participants (ACT self-help group: 79.7%; wait-list control group: 80.3%) completed the evaluations during T2 and 53.1% of the ACT self-help condition completed measures at T3. Figure 1 illustrates the flow of participants during this trial.

### Sociodemographic and clinical characteristics of the sample

At baseline, the women comprised 81.5% of the sample, with a mean age of nearly 51 years. The sample were mostly white/Caucasian (95%). Slightly more than a third were full-time (24.6%) or part-time (13.1%) employees, 43.1% were unemployed, and 18.5% were declared invalid. Almost two thirds of the individuals were living with a partner (65.1%) and 26.4% lived alone. Almost half (47.7%) had completed university studies. Just over 41% of participants had been suffering from CP for more than 10 years. The average intensity of the pain felt during the last week was evaluated at 5.8 on a scale of 0 to 10 (ACT self-help group: 5.7; wait-list control group: 5.9) where 0 was considered no pain. Fibromyalgia was the most frequent diagnosis encountered (38.5%), followed by back pain (20%) and neuropathic pain (16.9%), and almost half (46.9%) had various types of pain. Nearly 18% of the sample declared having been diagnosed with a depressive disorder, 16.9% with an anxiety disorder, and 7.7% of participants received more than one psychological diagnosis. As shown in Table 3, participants of the ACT self-help and wait-list control groups appear comparable in terms of their sociodemographic and clinical characteristics.

**Table 3.** Sociodemographic and clinical sample profile of the participants.^a^

	Total (N = 130)		ACT group (n = 64)		Control group (n = 66)	
Characteristics of the participants	Mean	SD	Mean	SD	Mean	SD
Age	51.06	12.67	51.91	14.21	50.21	11.01
Pain intensity on average in the past 7 days at baseline (T1) (evaluated on a scale of 0 to 10)	5.43	1.59	5.72	1.56	5.94	1.41
	Frequency	%	Frequency	%	Frequency	%
Sex						
Female	106	81.50	54	84.40	52	78.80
Male	24	18.50	10	15.60	14	21.20
Ethnicity						
White/Caucasian	124	95.40	63	98.40	62	93.94
Black	2	1.5	—	—	2	3.03
Aboriginal/First Nations/Metis	1	0.8	1	1.60	—	—
Others	1		—	—	1	1.50
Missing value(s)	2	1.5	—	—	2	3.03
Occupational status						
Working full time	32	24.62	14	21.90	18	27.30
Working part time (≤20 h/wk)	17	13.08	11	17.20	6	9.10
Unemployed	56	43.08	30	46.90	26	39.40
Declared invalid	24	18.46	8	12.50	16	24.20
Missing value(s)	1	0.77	1	1.60	—	—
Education level						
Primary education	1	0.77	1	1.60	—	—
Secondary education	16	12.31	8	12.50	8	12.10
Professional studies (professional diploma)	20	15.38	12	18.80	8	12.10
Collegiate studies/Cegep	31	23.85	16	25.00	15	22.70
University studies	62	47.69	27	42.20	35	53.00
Living situation						
Alone	34	26.36	11	17.20	23	34.80
With a partner	84	65.12	46	71.90	38	57.60
With a roommate	1	0.78	1	1.60	—	—
With parents	1	0.78	1	1.60	—	—
Others	9	6.98	5	7.80	4	6.10
Missing value(s)	1	0.78	—	—	1	1.50
Family income (gross)						
Less than $20 000	19	14.62	8	12.50	11	16.70
Between $20 000 and $39 999	25	19.23	13	20.30	12	18.20
Between $40 000 and $59 999	37	28.46	20	31.30	17	25.80
Between $60 000 and $79 999	18	13.85	7	10.90	11	16.70
Between $80 000 and $99 999	18	13.85	8	12.50	10	15.20
More than $100 000	13	10.00	8	12.50	5	7.60
Number of years living with daily pain						
Less than a year	3	2.33	3	4.70	—	—
Between 1 and 3 years	13	10.08	5	7.80	8	12.10
Between 3 and 5 years	20	15.50	11	17.20	9	13.60
Between 5 and 10 years	40	31.01	16	25.00	24	36.40
More than 10 years	53	41.09	28	43.80	25	37.90
Missing value(s)	1	0.80	1	1.60	—	—
Formal chronic pain diagnosis						
Yes	121	94.53	57	89.10	64	97.00
No	7	5.47	5	7.80	2	3.00
Missing value(s)	2	1.50	2	3.10	—	—
If so, which one(s)						
Headaches (migraines)	7	5.40	3	4.70	4	6.10
Fibromyalgia	50	38.50	25	39.10	25	37.90
Back pain	26	20.00	12	18.80	14	21.20
Cervical pain	6	4.60	3	4.70	3	4.50
Neuropathic pain	22	16.90	10	15.60	12	18.20
Arthritis	2	1.50	2	3.10	—	—
Disease-related pain (e.g., diabetes, cancer)	4	3.10	2	3.10	2	3.00
Others	10	7.69	5	7.80	5	7.60
More than one type one pain	61	46.92	31	48.44	30	45.45
Missing value(s)	3	2.31	2	3.10	1	1.50
Use of pain medication at intake						
Yes	112	86.15	57	89.10	55	83.30
No	17	13.08	7	10.90	10	15.20
Missing value(s)	1	0.77	—	—	1	1.50
Recent psychological diagnosis						
Yes	56	43.08	27	42.20	29	43.90
No	74	56.92	37	57.80	37	56.10
Which one(s)						
Adaptation disorder	8	6.20	3	4.70	5	7.60
Anxiety disorders	22	16.90	10	15.60	12	18.2
Depression	23	17.70	12	18.80	11	16.70
Substance abuse (alcohol, drugs)	2	1.50	1	1.60	1	1.50
Others	3	2.31	1	1.60	2	3.00
Multiple diagnoses	10	7.69	5	7.81	5	7.58
Currently in psychotherapy for pain						
Yes	23	17.69	10	15.60	13	19.70
No	107	82.31	54	84.40	53	80.30

^a^Given the missing data, some percentages do not add up to 100%.

ACT = acceptance and commitment therapy.

### Descriptive statistics

Table 4 presents the means observed between T1 and T2 on each variable for the ACT self-help program and wait-list control groups, including the standard deviations, confidence intervals, and range of scores for each measure.

**Table 4.** Means, standard deviations, and confidence intervals on the variables studied at T1 and T2 in the ACT self-help and wait-list groups.

		ACT self-help					Wait-list control					
					95% CI					95% CI		
Dependent variable	Measurement time	Mean	SD	*N*	Inf.	Sup.	Mean	SD	*N*	Inf.	Sup.	Range of scores
Pain-related disability (BPI)	T1	54.02	19.91	64	49.17	58.87	56.88	19.33	66	52.11	61.66	0–100
	T2	43.66	22.19	64	38.53	48.81	54.81	19.33	66	49.75	59.88	
Depressive symptoms (BDI-SF)	T1	10.71	6.31	64	9.28	12.14	9.44	5.19	64	8.01	10.87	0–39
	T2	8.61	5.97	64	7.21	10.02	9.42	5.35	64	8.02	10.82	
Pain acceptance (CPAQ)	T1	22.49	6.53	64	20.76	24.22	19.56	7.40	66	17.86	21.26	0–48
	T2	26.56	7.32	64	24.72	28.41	20.24	7.60	66	18.42	22.06	
Psychological inflexibility in pain (PIPS)	T1	56.34	10.23	64	53.70	58.99	59.24	11.15	65	56.61	61.87	7–84
	T2	49.25	13.05	64	46.26	52.24	59.18	11.07	65	56.22	62.15	

ACT = acceptance and commitment therapy; CI = confidence interval; Inf. = Inferior; Sup. = Superior; BPI = Brief Pain Inventory; BDI-SF = Beck Depression Inventory–Short Form; CPAQ = Chronic Pain Acceptance Questionnaire; PIPS = Psychological Inflexibility in Pain Scale.

### Pain-related disability (primary outcome variable)

**Figure 2.**
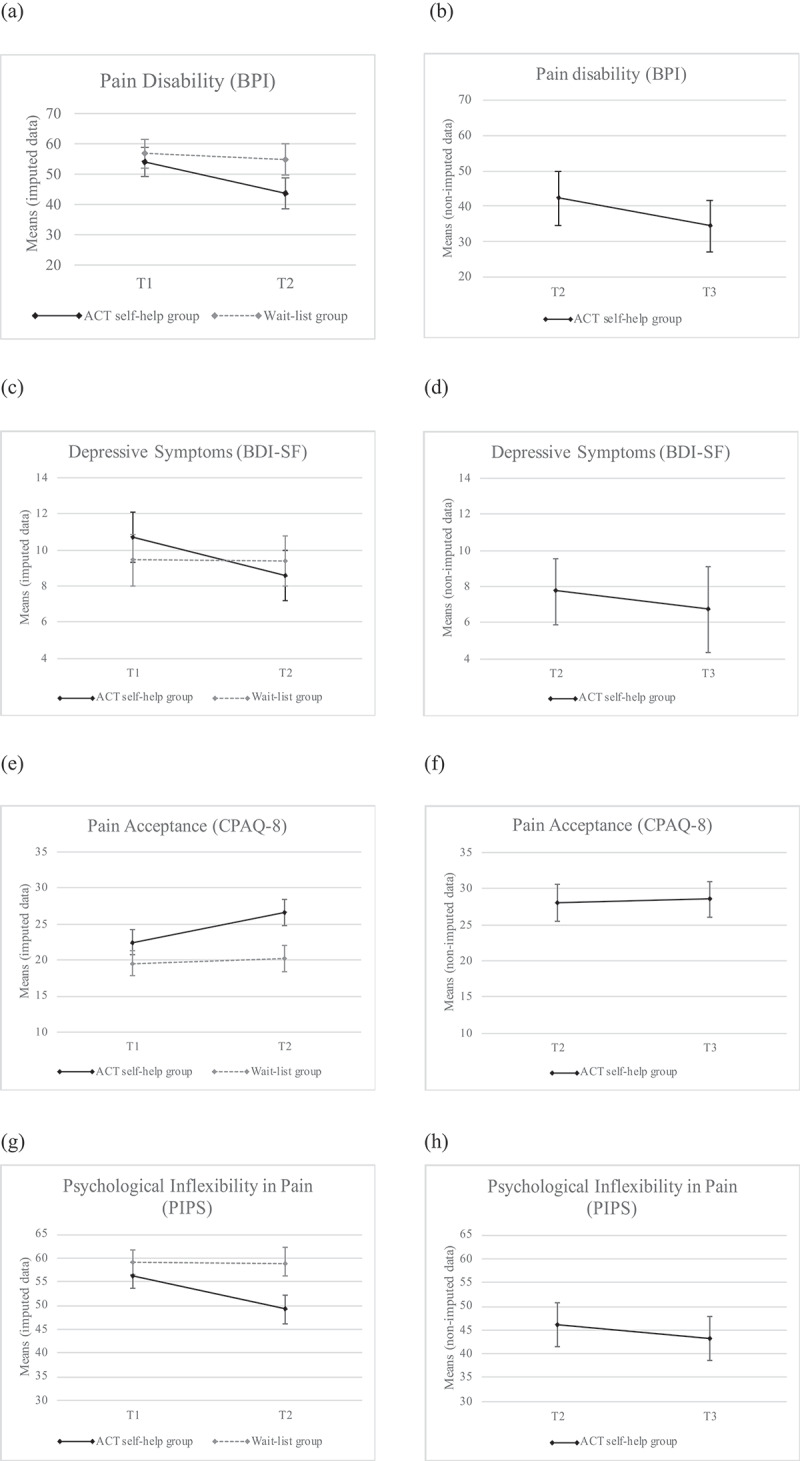
Graphic representations of the mean scores on each variable studied between T1 and T2 for the ACT self-help and wait-list groups (left panel) and between T2 and T3 for the ACT self-help condition (right panel).

Visual examination of the graph with the group marginal means for T1 and T2 presented in Figure 2a suggests an interaction effect between the time and group factors during T2 regarding pain-related disability (BPI). Table 5 presents the results of the statistical tests confirming this medium size (η^2^_partial_ = 0.074) interaction effect, *F*_(1, 128)_ = 10.21, *P* = 0.002. Analysis of simple main effects does indeed show a significant reduction in pain-related disability means for the ACT self-help group at T2, *F*_(1, 63)_ = 20.58, *P* < 0.001, η^2^_partial_ = 0.25, but not for the wait-list control condition, *F*_(1, 65)_ = 2.63, *P* = 0.110.

**Table 5.** Results of the analyses of variance and effect sizes of the differences between the ACT self-help and wait-list control groups at T1 and T2.^a^

	Simple main effects linked to treatment					Simple main effects linked to time					Interaction effects Group × Time				
Dependent variable	Measurement time	*F*	ddl	*P*	η^2^_partial_	Group	*F*	ddl	*P*	η^2^_partial_	*F*	ddl	*P*	η^2^_partial_	Cohen’s *d*
Pain-related disability (BPI)	T1	0.69	1, 128	0.407	0.01	ACT	20.58	1, 63	0.001*	0.25	10.21	1, 128	0.002*	0.07	0.57
	T2	9.35	1, 128	0.003*	0.07	Control	2.63	1, 65	0.110	0.04					
Depressive symptoms (BDI-SF)	T1	1.54	1, 126	0.217	0.01	ACT	10.87	1, 63	0.002*	0.15	6.59	1, 126	0.011*	0.05	0.46
	T2	1.17	1, 128	0.810	0.01	Control	0.001	1, 63	0.971	0.00					
Depressive symptoms (BDI-SF, excluding outliers)	T1	2.29	1, 124	0.133	0.02	ACT	13.91	1, 61	0.000*	0.19	7.70	1, 124	0.006*	0.06	0.50
	T2	1.09	1, 126	0.298	0.01	Control	0.04	1, 63	0.838	0.00					
Pain acceptance (CPAQ)	T1	5.71	1, 128	0.018	0.04	ACT	47.30	1, 63	0.001*	0.43	19.85	1, 128	0.001*	0.13	0.79
	T2	23.29	1, 128	0.001*	0.15	Control	2.00	1, 65	0.162	0.03					
Psychological inflexibility in pain (PIPS)	T1	2.36	1, 127	0.127	0.02	ACT	41.13	1, 64	0.001*	0.40	24.64	1, 127	0.001*	0.16	0.88
	T2	22.77	1, 128	0.001*	0.15	Control	0.004	1, 64	0.951	0.00					

*Significant results *P* ≤ 0.0167 (three comparisons made = simple main effects of time, group, and interaction).

ACT = acceptance and commitment therapy; df = degrees of freedom; BPI = Brief Pain Inventory; BDI-SF = Beck Depression Inventory–Short Form; CPAQ = Chronic Pain Acceptance Questionnaire; PIPS = Psychological Inflexibility in Pain Scale.

### Depression (secondary outcome variable)

Visual inspection of Figure 2c also suggests an interaction effect of the time and group factors on the depression variable (BDI-SF). However, the normality assumption and equality of the covariances could not be demonstrated for the depression variable (BDI-SF) for T1. After doing a square root transformation of the collected data for both time periods, conditions relative to the completion of a variance analysis were met. Nevertheless, although the result of this test is very close to the significance threshold, *F*_(1, 126)_ = 5.79, *P* = 0.018, it does not allow for the conclusion of an interaction effect. Only a main effect for time is observed, *F*_(1, 126)_ = 7.08, *P* = 0.009, η^2^_partial_ = 0.053 (see Table 5); however, by removing data from two outliers that are abnormally below average (two values >−3 SD), we obtain a significantly statistical interaction effect where almost 6% of the reduction of depressive symptoms are explained by the effect of the intervention, *F*_(1, 124)_ = 7.70, *P* = 0.006, η^2^_partial_ = 0.058, which can be considered a medium effect. Analysis of simple effects suggests that there is no statistically significant difference between the groups at T1, *F*_(1, 124)_ = 2.29, *P* = 0.133, or T2, *F*_(1, 126)_ = 1.09, *P* = 0.298; however, within the ACT self-help group, the mean difference between T1 and T2 demonstrates a significant effect, *F*_(1, 61)_ = 13.91, *P* < 0.001, η^2^_partial_ = 0.186, which is not the case for the wait-list control group, *F*_(1, 63)_ = 0.04, *P* = 0.838.

In sum, the results suggest an interaction effect between the time and condition factors, implying a significant reduction of depressive symptoms following the ACT self-help program with a medium effect (while controlling the data), which is not the case for the wait-list control group, for whom the BDI-SF scores remained unchanged.

### Pain acceptance (process variable)

Results for pain acceptance indicate an interaction effect of the time and group factors (see Figure 2e), with an increased average pain acceptance among ACT self-help participants postintervention, *F*_(1, 128)_ = 19.85, *P* < 0.001, η^2^_partial_ = 0.134. Although analyses demonstrate the presence of a statistically significant difference between the two groups at T1, *F*_(1, 128)_ = 5.71, *P* = 0.018, there is a significant increase in the scores between the T1 and T2 for the ACT self-help group, *F*_(1, 63)_ = 47.3, *P* < 0.001, η^2^_partial_ = 0.429, whereas no change is observed for the wait-list control group, *F*_(1, 65)_ = 2.00, *P* = 0.162.

### Psychological Inflexibility in Pain (process variable)

The ANOVA results obtained for the PIPS showed a significant interaction effect between the group and time factors, *F*_(1, 127)_ = 24.64, *P* < 0.001, η^2^_partial_ = 0.162, indicating a statistically significant reduction in psychological inflexibility related to pain for the ACT self-help group. Furthermore, the simple main effect analysis confirms a reduction in mean scores between T1 and T2 for the ACT self-help group, *F*_(1, 64)_ = 41.13, *P* < 0.001, η^2^_partial_ = 0.395, whereas there was no change between these two time periods for the wait-list control group, *F*_(1, 64)_ = 0.004, *P* = 0.951 (see Figure 2g, which illustrates the interaction effect on the time and group factors for the psychological inflexibility in pain variable).

### Effects of the intervention at 3-month follow-up

Table 6 shows a comparison of the mean scores of the ACT self-help group after the intervention (T2) and 3 months later (T3). Results of the paired *t* test indicate a statistically significant reduction on the primary outcome; that is, pain-related disability, *t*_(27)_ = 2.481, *P* = 0.020, with a mean score difference of 7.86 (SD = 16.76, 95% confidence interval [CI], 1.36, 14.36) between T2 and T3, with a small effect size (*d* = 0.47). For the depression variable, the normality condition (Shapiro-Wilk’s test, *P* < 0.001) was not met between T2 and T3 due to the presence of two outliers. Given the small sample size (*n* = 30), analyses were conducted with and without these values to verify the impact of these scores on the results. With outliers, there is no statistically significant difference between mean scores at posttest and follow-up, *t*_(29)_ = 0.98, *P* = 0.34. In other words, the benefits of the intervention condition are maintained; however, when we removed the two outliers from the analysis, the results suggest a small but statistically significant reduction in depressive symptomatology, *t*_(27)_ = 2.28, *P* = 0.031, with a mean score difference of 1.46 (SD = 3.39, 95% CI, 0.15, 2.78) and a small effect size (*d* = 0.43). The results on the pain acceptance measure showed no significant difference between T2 and T3, *t*_(31)_ = − 0.69, *P* = 0.49. However, the effects of the treatment seem to evolve positively in regards to psychological inflexibility, *t*_(30)_ = 2.26, *P* = 0.031, with the scores suggesting a statistically significant mean difference of 2.9 (SD = 7.16; 95% CI, 0.28, 5.53) between T2 and T3 with a small effect size (*d* = 0.41). The graphs (b, d, f, h) in Figure 2 show the maintenance of intervention improvements between T2 and T3.

**Table 6.** Results of paired *t* tests used to compare the results obtained at T2 and T3 in the ACT self-help group.^a^

					Difference of paired means							
							95% CI					
Dependent variable	Measurement time	Mean	SD	*N*	Mean	SD	Sup.	Inf.	*t*	ddl	*P* (bilateral)	Cohen’s *d* between T2 and T3
Pain-related disability (BPI)	T2	42.25	19.66	28								
	T3	34.39	19.05	28	7.86	16.76	1.36	14.36	2,48	27	0.020*	0.47
Depressive symptoms (BDI-SF)	T2	7.77	4.95	30								
	T3	6.73	6.46	30	1.03	5.77	−1.12	3.19	0.980	29	0.335	0.18
Pain acceptance (CPAQ)	T2	28.06	6.91	32								
	T3	28.53	6.66	32	−0.47	3.83	−1.85	0.91	−0.693	31	0.494	−0.12
Psychological inflexibility in pain (PIPS)	T2	46.00	12.50	31								
	T3	43.10	12.58	31	2.90	7.16	0.28	5.53	2,26	30	0.031*	0.41

*Significant results *P* ≤ 0.05.

ACT = acceptance and commitment therapy; CI = confidence interval; Sup. = Superior; Inf. = Inferior; df = degrees of freedom; BPI = Brief Pain Inventory; BDI-SF = Beck Depression Inventory–Short Form; CPAQ = Chronic Pain Acceptance Questionnaire; PIPS = Psychological Inflexibility in Pain Scale.

In terms of PGIC, an average of almost 54% of the participants from the ACT self-help group reported an improvement in their physical and mental health (pain, functioning, quality of life, and psychological well-being), approximately 23% deemed that it remained unchanged, and a little less than 24% reported that their health had deteriorated at T3. Lastly, the group reported a mean percentage of pain relief of 27.6% at T3 (minimum score: 0%; maximum score: 80%).

### Negative effects and adherence to treatment

Self-reported data on negative effects related to the intervention and adherence to treatment are presented in Table 7.

**Table 7.** Self-reported data on negative effects related to the intervention and adherence to treatment.^a^

Negative effects related to the intervention	Frequency	%
“I observed negative side effects following the intervention (e.g., intense pain, discomfort, etc.).”		
Strongly disagree	22	46.8
Disagree	18	38.3
Neutral	5	10.6
Agree	2	4.3
Strongly agree	0	0
“Certain exercises brought me negative side effects.”		
Strongly disagree	21	42
Disagree	18	36
Neutral	8	16
Agree	3	6
Strongly agree	0	0
“Meditation exercises brought me negative side effects.”		
Strongly disagree	24	47.1
Disagree	16	31.4
Neutral	6	11.8
Agree	5	9.8
Strongly agree	0	0
Adherence to treatment	Mean	SD
In which proportion have you read the book (0% to 100%)?	89.4	17.8
In which proportion have you used the participant’s book (0% to 100%)?	46.3	35.3
In which proportion have you used the links to the website to see or listen to audio recordings (0% to 100%)?	75.0	29.3
In which proportion have you put into practice the proposed exercises in the e-mails and in the book (0% to 100%)?	71.6	26.8
In which proportion have you put into practice the meditation exercises proposed in the e-mails and the book (0% to 100%)?	73.3	26.3

^a^There is a large portion of missing data (>50%). The percentages presented are based on data obtained.

## Discussion

The purpose of this study was to evaluate the efficacy of a self-help bibliotherapy-type psychological intervention program, based on ACT and with minimal support, for the management of chronic pain among adults from the community. It also aimed to bring additional empirical support to ACT in this field of expertise.

Our results suggest that the intervention had a greater effect than the wait-list control condition on pain-related disability immediately after the intervention (T2), with a medium effect size (*d* = 0.57). Furthermore, the IMMPACT expert consensus deems that a reduction of more than 1 point (scale of 0 to 10) for the different items of the BPI Interference subscale represents a clinically significant change.^50^ This study found a mean reduction of 1.04 point for the ACT self-help group between the T1 and T2, and this difference increased to 1.82 points between T1 and T3. These results are therefore very encouraging, because they suggest bibliotherapy can lead to a clinically significant improvement in pain-related disability.

As for depressive symptoms, the analyses revealed that the intervention led to a reduction of symptoms at posttest with a medium effect size (*d* = 0.5), when excluding two outliers from the analysis. Furthermore, according to BDI-SF norms,^41^ depressive symptoms would have decreased from T1 and T3, going from moderate to mild (see Tables 4 and 6). It seems that when linked to a person’s values, behavioral activation may have a positive effect on the mood of people with CP. Moreover, there is literature to support this intervention strategy to foster psychological well-being among various clinical and nonclinical populations.^51,52^

Regarding the variables linked to the intervention model, our results between T1 and T2 indicate that ACT, in a self-help bibliotherapy format, can help improve participants’ level of acceptance, with a medium effect size (*d* = 0.79). Our results also showed that this intervention helped reduce pain-related psychological inflexibility with a large effect size at T2 (*d* = 0.88). Overall, it seems that these two variables play a key role in the management of CP.^42,53,54^

The results of this study also suggest the maintenance or improvement of gains over a longer period of time, although the absence of control group data at T3 makes this suggestion speculative. At the 3-month follow-up, the scores of participants in the ACT self-help group showed further reduction in pain-related disability, depressive symptoms (results without two outliers), and psychological inflexibility with small effect sizes between T2 and T3, and the improvements on pain acceptance between T2 and T3 were maintained. However, this conclusion remains uncertain due to the absence of waiting list condition data at T3 and the high attrition rate of the ACT condition (nearly 50%) at this measurement time. It is also possible that these results are attributable to the lack of statistical power (*n* = 34).

Nevertheless, these results concur with previous studies in which ACT interventions for CP in a self-help format were shown to be effective.^15–19^ With regards to the PGIC results, approximately half of ACT self-help participants who were retained at 3-month follow-up reported that the intervention helped to improve their pain, physical functioning, quality of life, and psychological well-being over the last 3 months with an average pain relief of nearly 30%. Although this measure provides relevant information pertaining to long-term effects on the patient’s life in the longer term, until now, only one study^17^ took this measure into account among research evaluating self-help ACT in the management of CP. Therefore, its inclusion in the current study and the positive results obtained are an important addition to the existing literature.

In sum, our results are consistent with those of a recent meta-analysis^6^ evaluating ACT in the management of CP among adult clinical populations and adults from the community, which included guided Internet (*n* = 2),^16,18^ individual face-to-face (*n* = 2), manual-based self-help (*n* = 1),^15^ and group ACT intervention (*n* = 6) studies, and it brings further support to the Web-based and manual-based formats. The analyses also demonstrated effect sizes that were superior or equivalent at posttreatment and longer term follow-up on pain-related-disability,^18^ depression,^17,18^ pain acceptance,^17^ and psychological flexibility^18^ in comparison with similar RCTs (population, self-administered intervention, and wait-list control group). Despite these positive results, the current study has certain limitations. First, the absence of an active comparison group limits generalization of the time effect and does not allow for the control of nonspecific variables or common factors. It would be worthwhile to compare this intervention program to an active control group being educated on pain with minimal therapeutic support, for example. Second, for ethical reasons, it was not possible to complete all phases of a reversed replicated design^55^ and to wait 3 months before starting the intervention in the wait-list control group. This type of design would have helped to evaluate the effects of the intervention over the longer term (3 months) for each group (experimental and control), compare results, and obtain a higher level of evidence to support the efficacy of the ACT self-help intervention. Third, in order to have a more realistic view of the evolution of participants who completed the intervention given the high attrition rate (nearly 50%), the long-term analysis was conducted without imputed data, which differed from the sample used for the medium-term analysis. Though we found the same results, this choice could be criticized by some researchers. Fourth, participants were recruited through an association of people with CP; whether people who join this type of association differ from those with CP in the general population who do not is unknown, but it is possible that they differ (e.g., in terms of support-seeking or motivation for treatment). Although participants reported relatively high levels of pain intensity at baseline (mean: 5.43/10; SD ± 1.59), it is unknown whether they differ from a clinical population seen in tertiary sectors of care in terms of their psychological functioning or motivation to commit to a self-help intervention program. Fifth, the results suggest improvements in terms of ACT therapeutic processes but only on two of six processes (pain acceptance and psychological inflexibility). According to McCracken and Vowles,^29^ one of the challenges of future research is to identify the action mechanisms and target components of ACT in the management of CP. In the future, the use of mediation analyses would be crucial to confirm that the effects observed postintervention (disability, mood) can be attributed to the improvements on these processes. Sixth, although the attrition rate at T2 is comparable to that found in similar studies evaluating self-administered psychological interventions (17.4%^56^; 21.3%^13^; 31%^35^), we do not know why participants dropped out. It would be worthwhile to evaluate whether these dropouts are due, for example, to a lack of support or motivation, nonacceptance of the intervention, or a difference in the participants’ feelings of self-efficacy in order to find concrete solutions to minimize attrition rates. Seventh, the assiduity with which participants completed the exercises proposed as part of the intervention, such as reading the book or completing the meditation exercises, was measured by self-report, thereby introducing potential bias. The use of more objective measures of adherence would have been challenging in the current context but would have been more reliable.

Despite these limitations, this study contributes incrementally to self-help psychological interventions in the management of CP. Until now, very few RCTs have evaluated the effectiveness of an ACT-based self-help intervention program for management of CP in the form of bibliotherapy^14,15^ and, more important, with minimal therapeutic contact. Not only does this trial further support this intervention method and type of therapeutic support, it also supports the fact that a self-help ACT treatment can be an effective and affordable solution to the obstacles related to the accessibility of face-to-face care for the management of CP in hospitals or tertiary centers.^57–59^ In a time when self-help is subject to various criticisms,^31–33^ more specifically, regarding the overestimation of the effectiveness of the bibliotherapies available to the general public based on ACT, this RCT uses a rigorous methodology to demonstrate the relevance of this treatment approach and treatment modality among adults with CP in the community. The current study included adults of different ages, male and female, and with various chronic pain diagnoses, which means that results can be applicable to a wide range of adults with CP. In the future, it would be worthwhile to identify profiles of patients who are most likely and least likely to benefit from this type of treatment, because this would be useful information to adequately use resources and make appropriate tailored recommendations for patients. It would also be helpful to further examine processes of change in such interventions in order to provide further support to the intervention model and to optimize the effectiveness of ACT in a self-administered format.^60^
